# Relating Rational and Experiential Thinking Styles With Trait Emotional Intelligence in Broader Personality Space

**DOI:** 10.5964/ejop.v15i1.1692

**Published:** 2019-02-28

**Authors:** Biljana Jokić, Danka Purić

**Affiliations:** a Center for Study in Cultural Development, Belgrade, Serbia; b Social Psychology Laboratory, Faculty of Philosophy, University of Belgrade, Belgrade, Serbia; c Department of Psychology, University of Belgrade, Belgrade, Serbia; d Laboratory for Research of Individual Differences, Faculty of Philosophy, University of Belgrade, Belgrade, Serbia; University of Belgrade, Belgrade, Serbia

**Keywords:** trait emotional intelligence (TEI), cognitive-experiential self-theory (CEST), rational experiential inventory (REI-40), thinking styles, HEXACO

## Abstract

The usual distinction between rational and intuitive thinking styles is still a subject of scientific debate, as there is no consensus about their nature, mutual relations and relations to other personality constructs. Cognitive-experiential self-theory (CEST) proposes rational and experiential thinking styles as original personality constructs not fully explainable by five-factor personality models. Following CEST, we aimed to examine: 1. The uniqueness of rational and experiential dimensions by relating them to other personality constructs: trait emotional intelligence (TEI) and HEXACO; 2. Thinking style profiles defined through combined rational and experiential dimensions, and the possible role of TEI in understanding them. A total of 270 undergraduate students (82% females) completed the TEIQue-SF, REI-40, and HEXACO-PI-R. Our results showed that constructs from all three paradigms were low to moderately correlated to each other. TEI had incremental validity in explaining both rational and experiential dimensions, but large amounts of their variances remained unexplained by both TEI and HEXACO. We revealed four thinking style profiles defined through combined rational and experiential dimensions. TEI was the highest when both dimensions were high and the lowest when both were low, which could be related to processes of understanding and managing emotional functioning – proposed as an essential part of TEI, while within CEST they are seen as the way in which rationality influences experientiality. This finding might be of specific significance for understanding irrationality as not exclusively related to high intuition, but to low rationality as well.

Many authors acknowledge reflective/analytic and intuitive/automatic thinking styles, recognizing individual differences in their habitual use as relatively stable tendencies throughout time and situations (Phillips, Fletcher, Marks, & Hine, 2016). There is strong empirical support for the assumption that these are two independent dimensions rather than the opposite ends of a bipolar continuum (Akinci & Sadler-Smith, 2013; Hodgkinson, Sadler-Smith, Sinclair, & Ashkanasy, 2009; Wang, Highhouse, Lake, Petersen, & Rada, 2017). This assumption is also in line with Cognitive-experiential self-theory (CEST) which provided one of the most often used instruments for measuring individual differences in thinking styles, the Rational-Experiential Inventory (REI) (Epstein, 2003, 2016; Pacini & Epstein, 1999). Moreover, CEST is proposed as a personality theory defining rational and experiential thinking styles as unique constructs, which cannot be reduced to or entirely explained by classical personality traits from the Big Five model.

In this study, we were interested in determining the relations between thinking styles and some other personality constructs, namely the six-factor personality model and trait emotional intelligence. In general, connecting different paradigms and testing uniqueness and mutual overlapping of constructs proposed to explain personality space could further our understanding of human behavior – in this particular case, one’s preference for rational or/and experiential thinking styles, and moreover preference for irrationality.

## Cognitive-Experiential Self-Theory

According to CEST, an experiential system (ES) is preconscious, automatic, effortless, rapid, and associated with affect, while a rational system (RS) is conscious, analytical, effortful, slower than experiential, and affect-free (Epstein, 2016)^i^. Each has its advantages and disadvantages, and it might be *reasonable* to use one over another depending on context (Epstein, 2010). The authors of CEST emphasized that their theory did not offer new insights about RS, but rather considered ES in a way different from the usual understanding of automatic/preconscious processes. ES is understood as an organized system with heuristics as primarily adaptive processes. This is essentially different from conceptualizing heuristics as mutually unrelated cognitive shortcuts for making decisions under uncertainty (as in Tversky & Kahneman, 1974), when people rely on so-called “subjective probabilities” – beliefs about the likelihood of events (e.g. outcomes of elections). ES is proposed as an alternative for the Freudian unconscious system, but unlike the maladaptive unconscious in psychoanalysis, ES is considered an adaptive learning system. In fact, both ES and RS are learning systems – ES learns from experience and outcomes, while RS learns through inference. The assumption is that both ES and RS have their own form of intelligence – the intelligence of RS corresponds to the constructs assessed by classical IQ tests, while the intelligence of ES is proposed as an original construct, assumed to include practical intelligence, social intelligence, and emotional intelligence, and an original instrument has been created for measuring it (Constructive Thinking Inventory, CTI) (Epstein, 1998, 2010).

The CEST assumption is that the experiential system is necessary for survival and evolutionarily much older than the rational one. However, ES is also proposed to be a source of superstitions, prejudices, and biases in reasoning, which is empirically supported (Aarnio & Lindeman, 2005; King, Burton, Hicks, & Drigotas, 2007; Stojanov, 2015). The influence of ES on RS could be automatic, outside of awareness, and mediated by feelings. The identification of these feelings is supposed to be a first and important step in controlling that influence (Epstein, 2003). The successfulness of this process may depend on the specific combination of experientiality and rationality dimensions, based on the assumption about their orthogonality. An empirical study that examined interrelated rationality and experientiality effects on several criterion variables revealed four thinking style profiles: rationally dominant (high rationality / low experientiality), experientially dominant (high experientiality / low rationality), dual preference (high experientiality / high rationality), and disengaged (low experientiality / low rationality). Moreover, rationally dominant participants had the lowest scores on a superstition scale – significantly lower than experientially dominant participants, with dual preference participants in-between (Fletcher, Marks, & Hine, 2012). Although this study confirmed the role of the experiential dimension in irrational thinking, it implied that the rational dimension was important as well. This is evidenced by the difference in superstition scores between the dual-preference and experientially dominant participants, as both groups had high experientiality scores, but differed in their preference for rationality.

## Thinking Styles in Relation to Classic Personality Traits

Since CEST is proposed as a personality theory, its authors tested how it fared by some classical personality models. They demonstrated that REI provides information not covered by classical five-personality trait topologies, e.g. Big Five as a predictor of REI explains only 37% of the variance of rationality and 11% of experientiality (Epstein, 2003; Pacini & Epstein, 1999). Besides the conclusion that REI and Big Five measure different constructs, CEST authors also stated that Big Five models provide information mainly related to the rational (or conscious) thinking style, while they cannot capture psychological constructs associated with automatic (preconscious) information processing. Similar results were reported cross-culturally in Dutch and Spanish samples, with even lower percentages of explained variance: 22% for rationality and 7% for experientiality, respectively, in the Dutch sample and 6% and 2% in the Spanish sample (Witteman, van der Bercken, Claes, & Godoy, 2009). However, with regards to specific relations of Big Five traits with rational and experiential dimensions, the results were not entirely consistent, e.g. in the Dutch, but not the American sample, rationality was positively correlated to Agreeableness, while a low correlation between the experiential dimension and Conscientiousness was positive in the American and negative in the Dutch sample.

Since results on all three samples are consistent in that both rational and experiential dimensions kept specific variance, beyond that overlapping with personality traits from classical five-factor models, we were interested in exploring a possible contribution of another personality construct to the understanding of thinking style dimensions. For this purpose, we chose trait emotional intelligence (TEI) or trait emotional self-efficacy, defined as a constellation of emotional perceptions assessed via questionnaires and rating scales (Petrides, Pita, & Kokkinaki, 2007).

## The Possible Role of Trait Emotional Intelligence in Understanding Thinking Styles

TEI has been established as a recognized construct, widely tested and empirically supported (Andrei & Petrides, 2013; Andrei, Siegling, Aloe, Baldaro, & Petrides, 2016; Lea, Qualter, Davis, Pérez-González, & Bangee, 2018; Petrides et al., 2016; Rubaltelli & Pittarello, 2018), although there has been a debate around both the term (emotional intelligence) and the sense of the construct (since it overlaps with classic personality traits).

Concerning the term *emotional intelligence* (EI), it is important to note that unlike the classical EI construct which represents a specific form of *ability,* TEI represents emotional *self-perceptions*. Critics argue that, because it does not represent intelligence in the common sense of the concept, TEI contributes to the confusion in EI literature (Mayer, Salovey, & Caruso, 2008). As previously mentioned, CEST proposes its own specific understanding of emotional intelligence as a form of ES intelligence: it is the intelligence of automatic thinking that underlies emotions (Epstein, 1998, 2016). Having in mind the different meanings of the concept emotional intelligence in all those paradigms, our aim was not to compare them in terms of their plausibility or advantages of one over another but to explore possible relations between TEI and both rational and experiential dimensions. Why should we expect TEI to be related to rational and experiential dimensions from CEST?

TEI has been introduced as a second order personality trait that incorporates emotion-laden characteristics which are mostly spread over different personality traits of the Big Five model (De Raad, 2005; Petrides & Furnham, 2001; Van der Linden, Tsaousis, & Petrides, 2012). TEI includes 15 facets (e.g. self-esteem, social awareness, empathy, emotion perception, emotion regulation, stress management, etc.), with 13 of them forming four factors (self-control, emotionality, sociability and wellbeing), and the remaining two (adaptability and self-motivation) contributing directly to the global TEI score (Andrei et al., 2016; Petrides, 2009). As such, TEI considerably overlaps with constructs from the Big Five taxonomy, especially with Neuroticism and Extraversion, as these dimensions are particularly emotion-laden (Saklofske, Austin, & Minski, 2003; Siegling, Furnham, & Petrides, 2015). However, the authors of TEI treat these high correlations (over .60 with Neuroticism and over .50 with Extraversion) as expected and theoretically incorporated in the model (De Raad, 2005; Petrides & Furnham, 2001; Van der Linden et al., 2012). Moreover, research has shown that TEI has unique contributions in predicting various psychological variables, beyond the variance explained by the Big Five model (Jolić-Marjanović & Altaras-Dimitrijević, 2014; Siegling, Vesely, Petrides, & Saklofske, 2015). Therefore, based on the nature of rationality and, even more so, experientiality, it would not be surprising if TEI had an incremental contribution in predicting these two constructs as well.

Namely, as a constellation of emotional self-perceptions, TEI could be expected to capture processes related to the experiential system from the CEST paradigm. CEST assumes that ES is closely associated with affect (influencing affect and being influenced by affect) and REI also includes self-report measures about ability and preference in relying on affect (e.g. *I trust my initial feelings about people; I tend to use my heart as a guide for my actions*). Additionally, a broad definition of TEI which incorporates various variables from the domains of social relations, self-control, or more general well-being, also corresponds to past findings from the CEST paradigm which showed that the intelligence of the experiential system (measured by CTI) was positively correlated with social competence, leadership ability, ability to cope with stress, emotional adjustment, physical wellbeing, etc. (Epstein, 2003).

However, it is possible that TEI is not exclusively related to the experiential dimension. TEI also includes self-perceptions about regulation and management of emotions, which implies an understanding of emotional functioning – from the CEST perspective, the rational dimension has an important role in that process by aiding the understanding of how the experiential system operates and by correcting it to have more appropriate initial reactions (Epstein, 2003, 2016).

To our knowledge, no empirical research has systematically related CEST rationality and experientiality with the TEI paradigm. A study that tested the relation between REI and self-reported emotional intelligence revealed that both experientiality and rationality positively relate to EI, but this study did not control for other personality traits, and more importantly, although the construct of EI was discussed as both a trait and an ability, the instrument for measuring it operationalized EI as an ability, rather than a trait (Schutte et al., 2010).

## Current Study

The general goal of the current study was to examine thinking style constructs from the CEST paradigm by relating them to TEI, while also taking into account the variance shared with basic personality traits. Unlike past studies which included Big Five models, we decided to employ a six-factor model, HEXACO. The specific goals of our study were: 1. Assessing the relations between the constructs from CEST, TEI, and HEXACO paradigms, with particular emphasis on the amount of thinking style variance explained by HEXACO, and in addition by TEI; 2. Identifying thinking style profiles through combined rational and experiential dimensions (as in Fletcher et al., 2012) and further exploring their relations to TEI.

### Relations Between Constructs

In order to determine the relations between REI, TEI, and HEXACO, we planned to establish zero-order correlations between these three personality domains. While HEXACO, to our knowledge, has not been empirically connected to REI so far, this model has already been tested in the TEI paradigm: TEI had a high positive correlation with eXtraversion, a moderate negative correlation with Emotionality, while correlations with other traits were all positive and low to moderate in intensity (Veselka et al., 2010).

It is important to note that HEXACO incorporates a large amount of variance not captured by five-factor models (Ashton & Lee, 2007; Lee & Ashton, 2018). This primarily refers to the inclusion of an additional, broad Honesty/Humility factor. However, the authors suggest that HEXACO may also have predictive advantages over five-factor models in the domain of Emotionality. Namely, HEXACO Emotionality and Agreeableness are rotational variants of Neuroticism and Agreeableness from the Big Five model, excluding some and including other domains. Having in mind the inconsistent past findings concerning the relations between Big Five personality traits and thinking styles (Pacini & Epstein, 1999; Witteman et al., 2009), we were particularly interested in testing REI in relation to HEXACO in order to get new insights into the nature and interrelations of these dimensions.

As our study is exploratory in nature, we did not have empirically based hypotheses regarding relations between thinking styles and TEI. However, based on the theoretical considerations above, we did expect some amount of overlap between trait emotional intelligence and both experientiality and rationality.

After testing for correlations between constructs, we planned to determine the percentage of variance of rational and experiential dimensions explained by HEXACO personality traits. Based on past findings of Big Five model contributions to explaining the variance of thinking style dimensions, we expected that personality traits would explain less than half of REI dimensions’ variance and that the variance explained will be higher for rationality than for experientiality (Pacini & Epstein, 1999).

Furthermore, we sought to examine the incremental validity of TEI in predicting rational and experiential dimensions. This would allow us to determine the amount of overlap between TEI and REI constructs after basic personality traits have been controlled for and, at the same time, test whether TEI, as a second-order personality trait, does indeed have a significant additional contribution in predicting other constructs. In other words, we could simultaneously test both TEI and CEST paradigm assumptions that the constructs they propose provide additional information to understanding personality space, beyond the explanation already provided by the classic personality models. We considered TEI as a predictor of REI (and not vice versa, which would also be plausible), only because our primary interest was related to possible predictors of thinking styles, and not of emotional intelligence.

### The Role of TEI in Understanding Thinking Style Profiles Defined Though Combined Rational and Experiential Dimensions

If rationality and experientiality truly are independent dimensions, simple correlation-based analyses may fail to capture more complex relationships between thinking styles and other variables. As Fletcher et al. (2012) have demonstrated, four thinking style profiles were differentially related to measures of superstition. Following this approach, we aimed to further explore the relation of TEI with thinking style profiles defined through combinations of rational and experiential dimensions. Based on the theoretical consideration of the expected positive relation between TEI and both rationality and experientiality, we expected that TEI would be most pronounced in the high rational / high experiential thinking profile, while it would be the least present in the low rational / low experiential profile. Intriguingly, a recent study showed that, in a clinical sample, TEI had a significant direct negative effect on irrational beliefs (measured by the irrational beliefs test; Petrides, Gomez, & Perez-Gonzalez, 2017). If we assume that TEI does consistently determine (or correlates with) irrationality, based on findings showing that high rationality / low experientiality profile was the least, and high experientiality / low rationality the most related to superstitions (Fletcher et al., 2012), it could also be hypothesized that TEI would follow this pattern.

## Method

### Participants and Procedure

A power analysis indicated that a sample of at least 211 participants is needed to detect a .20 correlation with a power of .90 (and the alpha probability level of .05). This number rises to 266 if the power is set at .95. Detecting an incremental contribution of a predictor of *R*^2^ = .05, after six other predictors have already been entered in the hierarchical regression, with the alpha probability .05 and power .90 requires 202 participants, or 249 if power is set to .95. As we did not have specific hypotheses regarding the latent profile analysis, we relied on the results of simulation studies that indicated sample size to have low relevance in detecting the true number of classes when the sample size is *N* > 100 or *N* > 200 (Wurpts & Geiser, 2014), as well as the Fletcher et al. (2012) study which identified four thinking style profiles with a sample size of *N* = 304.

The present study was conducted on a sample of 270 students from the Department of Psychology at the University of Belgrade (82% females, average age *M* = 21.3, *SD* = 2.3). The high percentage of females in the sample reflects the gender structure of the population of psychology students at Belgrade University.

The research was conducted in accordance with the Declaration of Helsinki, as well as the Serbian Psychological Society ethical guidelines. Students were invited to take part in the study in exchange for partial course credit. The questionnaires were administered online, with about half of the students completing the battery from their home and others completing it in a computer classroom at the university, as part of their regular classes. All students provided informed consent before filling in the questionnaires and alternative course credit awarding activities were offered to those who did not wish to participate.

### Instruments

#### HEXACO Personality Inventory-Revised

The HEXACO Personality Inventory-Revised (HEXACO-PI-R; Ashton & Lee, 2007) assesses six personality dimensions: Honesty/Humility (H), Emotionality (E), eXtraversion (X), Agreeableness (A), Conscientiousness (C) and Openness to Experience (O). We employed the Serbian translation of the 100-item version of this scale (Međedović, Čolović, Dinić, & Smederevac, 2017). Participants responded by indicating their level of agreement with each statement on a 5-point Likert scale (1 = strongly disagree, 5 = strongly agree). Cronbach’s alpha reliabilities of dimensions were satisfactory, ranging from .78 for Emotionality to .84 for Conscientiousness for the Serbian version of the scale and the factorial structure of the Serbian version of the instrument corresponded to the original (Međedović et al., 2017).

#### Rational-Experiential Inventory-40

The Rational-Experiential Inventory-40 (REI-40; Pacini & Epstein, 1999) is a 40-item self-report instrument measuring rational and experiential thinking styles. We used the Serbian translation of this scale (Branković, 2012). Responses are given on a 5-point Likert scale (1 = definitely not true of myself to 5 = definitely true of myself). Apart from the two higher-order scores, the rational and the experiential thinking style, four lower-order factors relating to self-perception of ability and preference of using these two thinking styles can also be calculated – Rational Ability (RA), Rational Engagement (RE), Experiential Ability (EA) and Experiential Engagement (EE). Both rationality and experientiality scales comprise 20 items, while RA, RE, EA, and EE are assessed by 10 items each. The original study obtained high rationality and experientiality scale Cronbach’s alpha reliabilities of .90 and .87, respectively, while subscale reliabilities ranged from .79 for EE to .84 for RE.

#### Trait Emotional Intelligence Questionnaire-Short Form

The Trait Emotional Intelligence Questionnaire-Short Form (TEIQue-SF; Petrides, 2009) is a 30-item scale assessing trait emotional intelligence. In our study, we used the Serbian translation of TEIQue-SF (Jolić-Marjanović & Altaras-Dimitrijević, 2014). Responses are given on a 7-point Likert scale (1 = completely disagree, 7 = completely agree). Apart from the global TEI score, the instrument provides scores for four TEI factors: well-being (6 items), self-control (6 items), emotionality (8 items) and sociability (6 items). TEIQue-SF was reliable for both male and female samples, with Cronbach’s alpha reliabilities of .84 and .89, respectively (Petrides & Furnham, 2006).

## Results

### Descriptive Statistics

With the exception of Openness, all variables were approximately normally distributed (Kolmogorov-Smirnov test did not indicate significant deviations from the normal distribution, Table 1). Considering that we had a young, student sample, the positively skewed and leptokurtic Openness distribution was not unexpected. The deviations from normality were, however, not large, so we decided to proceed with parametric analyses of the data. Cronbach alpha reliabilities were very good for all scales. Only Rational Ability subscale reliability was slightly below .8, but considering that this subscale has only 10 items, the obtained value is satisfactory.

**Table 1:** Descriptive Statistics for all Used Measures

Measure	*N*	*M*	*SD*	Min	Max	Sk	Ku	KSD	α
H	248	3.60	0.63	1.38	4.88	-.56	.09	.08	.83
E	248	3.43	0.64	1.56	4.94	-.21	-.16	.05	.84
X	248	3.36	0.77	1.31	4.88	-.44	-.26	.07	.91
A	248	3.03	0.66	1.31	4.69	-.04	-.40	.06	.86
C	248	3.68	0.63	2.06	5.00	-.13	-.53	.05	.86
O	248	3.84	0.58	1.55	5.00	-.95	1.76	.09*	.83
RD	268	3.86	0.55	2.00	5.00	-.32	.03	.06	.88
RA	268	3.84	0.56	2.10	5.00	-.25	-.30	.06	.78
RE	268	3.88	0.64	1.00	5.00	-.60	.94	.06	.83
ED	268	3.30	0.61	1.50	4.65	-.16	-.29	.04	.91
EA	268	3.38	0.66	1.70	4.90	-.03	-.46	.04	.85
EE	268	3.14	0.70	1.20	4.89	-.29	-.22	.07	.85
TEI	270	5.09	0.74	2.67	6.70	-.52	.35	.06	.89

*Note*. Sk = Skewness; Ku = Kurtosis; KSD = Kolmogorov-Smirnov most extreme absolute difference; α = Cronbach’s alpha reliability coefficient; H = Honesty/Humility; E = Emotionality; X = eXtraversion; A = Agreeableness; C = Conscientiousness; O = Openness to Experience; RD = Rational thinking style dimension; RA = Rational Ability; RE = Rational Engagement; ED = Experiential thinking style dimension; EA = Experiential Ability; EE = Experiential Engagement; TEI = trait emotional intelligence.

*p < .05.

### Relations Between Constructs

As expected, HEXACO traits were mostly unrelated to one another with the exception of a moderate positive correlation of Honesty/Humility and Agreeableness, a small positive correlation of Honesty/Humility and Conscientiousness and a small negative correlation of Openness to Experience and Emotionality (Table 2). In line with CEST assumptions, experiential and rational dimensions were uncorrelated, while ability and engagement subdimensions within rationality and experientiality were highly correlated both with one another, as well as with their superordinate dimensions (to an even higher degree). Rationality and its subdimensions showed low to moderate correlations with eXtraversion, Conscientiousness and Openness traits from the HEXACO model. Rational Ability had a small negative correlation with Emotionality, while Rational Engagement had a small positive correlation with Honesty/Humility. Experientiality, on the other hand, had no significant correlations with either of the HEXACO traits, save for a small negative correlation between Experiential Engagement and Conscientiousness. TEI had a strong positive correlation with eXtraversion, and low positive correlations with Agreeableness and Conscientiousness, which is in line with theoretical expectations. It also correlated positively with both rationality and experientiality and their subdimensions, although correlations with rationality were in the moderate range, while those with experientiality were low.

**Table 2:** Correlations of HEXACO, REI and TEI Dimensions

Measure	H	E	X	A	C	O	RD	RA	RE	ED	EA	EE
E	.09											
X	-.07	-.06										
A	.31**	-.04	.03									
C	.15*	.04	.09	.07								
O	-.06	-.14*	.02	-.10	-.07							
RD	.11	-.12	.24**	-.02	.26**	.41**						
RA	.01	-.15*	.22**	-.03	.25**	.26**	.90**					
RE	.18**	-.08	.21**	-.01	.22**	.46**	.92**	.66**				
ED	-.05	.07	.11	-.06	-.10	.07	-.09	-.14*	-.02			
EA	-.07	.04	.08	-.12	-.03	.07	-.04	-.07	-.01	.92**		
EE	-.03	.09	.12	.00	-.16*	.06	-.11	-.19**	-.03	.93**	.69**	
TEI	.05	-.06	.61**	.15*	.21**	.01	.37**	.35**	.32**	.19**	.17**	.17**

*Note*. H = Honesty/Humility; E = Emotionality; X = eXtraversion; A = Agreeableness; C = Conscientiousness; O = Openness to Experience; RD = Rational thinking style dimension; RA = Rational Ability; RE = Rational Engagement; ED = Experiential thinking style dimension; EA = Experiential Ability; EE = Experiential Engagement; TEI = trait emotional intelligence.

*p < .05. **p < .01.

### HEXACO and TEI as Predictors of Rational and Experiential Dimensions

In order to evaluate whether HEXACO traits were significant predictors of rational and experiential thinking styles, as well as whether TEI had an incremental contribution in prediction, we used the hierarchical method of linear regression. Rationality and experientiality were used as dependent variables, HEXACO traits were entered as predictors in the first step of the analyses and TEI was added in the second step. As shown in Table 3, rationality was significantly predicted by HEXACO personality traits, namely Honesty, eXtraversion, Conscientiousness, and Openness, while this was not the case for Emotionality. TEI, on the other hand, was a significant predictor of both rationality and experientiality, even after accounting for the variance explained by HEXACO traits. It is also interesting to note that eXtraversion was no longer predictive of rationality after TEI was entered in the regression, indicating that even though there is a substantial overlap between eXtraversion and TEI, TEI may be a more useful construct in predicting certain specific traits and behaviors.

**Table 3:** Results of the Hierarchical Regression Analyses Predicting Rationality and Experientiality by HEXACO Traits and TEI

Predictor	Standardized regression weights			
	Rational thinking style		Experiential thinking style	
	Step 1	Step 2	Step 1	Step 2
Honesty/Humility	.14*	.13*	-.02	-.03
Emotionality	-.08	-.07	.09	.10
eXtraversion	.22***	.03	.12	-.05
Agreeableness	-.05	-.08	-.04	-.08
Conscientiousness	.25***	.21***	-.11	-.15
Openness	.41***	.41***	.07	.07
TEI		.30***		.29**
*F*(*df*)	17.78 (6,239)***	19.26 (7,238)***	1.66 (6,239)	3.34 (7,238)**
*R*^2^ change	.31***	.05***	.04	.05**

*Note*. TEI = trait emotional intelligence.

*p < .05. **p < .01. ***p < .001.

In order to account for the gender disbalance in our sample, we ran two three-step hierarchical analyses, with rationality and experientiality as criterion variables and binary-coded gender as the first-step predictor, adding HEXACO traits in the second and TEI in the third step. Gender was, however, not a significant predictor of either rationality or experientiality in either step of the analyses, so we excluded it from the final model for clarity. We also ran the analyses using TEI facets as predictors in the second step, instead of the total score. The percentage of explained variance was essentially the same, so we kept global TEI for further analysis.

### TEI and Combined Thinking Style Dimensions: Latent Profile Analysis

In order to assess the relationship of TEI with the combination of rationality and experientiality, we employed a latent profile analysis (LPA). The aim of LPA is to identify latent groups in the data, thereby explaining the manifest correlations of continuous variables (Oberski, 2016). In other words, when running a latent profile analysis, we assume that the correlations between manifest variables can (fully, or to a certain degree) be explained by the fact that participants belong to one of several latent groups, which differ in their scores on the manifest variables. As previous research had demonstrated that thinking style profiles can be readily identified (Fletcher et al., 2012), we were particularly interested in determining, via LPA, whether TEI was differentially pronounced in these thinking style profiles.

The analysis was done using the tidyLPA R package (Rosenberg, Schmidt, Beymer, & Steingut, 2018). All solutions were fitted under the so-called Model 1 (Table 4), which assumes varying means between groups and equal variances, while variable covariances within groups are fixed to zero. Other models differ with respect to how they model group variances (equal or varying) and covariances between groups (set to zero, equal or varying).

**Table 4:** Possible Model Specifications in Latent Profile Analysis

Parameter	Model 1	Model 2	Model 3	Model 4	Model 5	Model 6
Means	Varying	Varying	Varying	Varying	Varying	Varying
Variances	Equal	Equal	Varying	Varying	Equal	Varying
Covariances	Zero	Equal	Zero	Equal	Varying	Varying

Model 1 is the most restrictive model, as it assumes that practically all correlations between variables can be explained by latent group membership, but was suitable for our data, considering the CEST assumption of experiential and rational dimensions’ orthogonality. Moreover, this model is considered to be the most parsimonious and some authors suggest that the use of other, more complex models should be justified by significant model fit improvements (Pastor, Barron, Miller, & Davis, 2007; Rosenberg et al., 2018).

We estimated a range of profile solutions, from two to six latent groups, using REI facets and TEI summary score as input variables. Despite not expecting REI ability and engagement facets to behave differently from one another based on correlations, including them in the analysis allowed for possible identification of more differentiated profiles than just low/high rationality/experientiality. We also tested a number of LPA models using only the rational and experiential dimensions. The results were essentially the same as reported here, but with lower entropy values for the whole range of solutions. Therefore, we interpreted the solutions obtained on REI subdimensions. Table 5 shows fit indices of all tested profile solutions. Good models are characterized by low AIC and BIC values and high Log-Likelihood and entropy values.

**Table 5:** Fit Indices for Tested Profile Solutions

Profile solution	LogLik	AIC	BIC	Entropy	Numbers in classes
2	1823.04	3678.08	3735.54	0.92	139, 129
3	1779.99	3603.98	3682.98	0.86	74, 85, 109
4	1746.57	3549.14	3649.68	0.85	47, 91, 64, 66
5	1722.63	3513.26	3635.35	0.85	25, 39, 81, 67, 56
6	1714.26	3508.52	3652.16	0.84	24, 38, 75, 70, 4, 57

*Note*. LogLik = Log Likelihood; AIC = Akaike’s Information Criterion; BIC = Bayesian Information Criterion.

**Figure 1:**
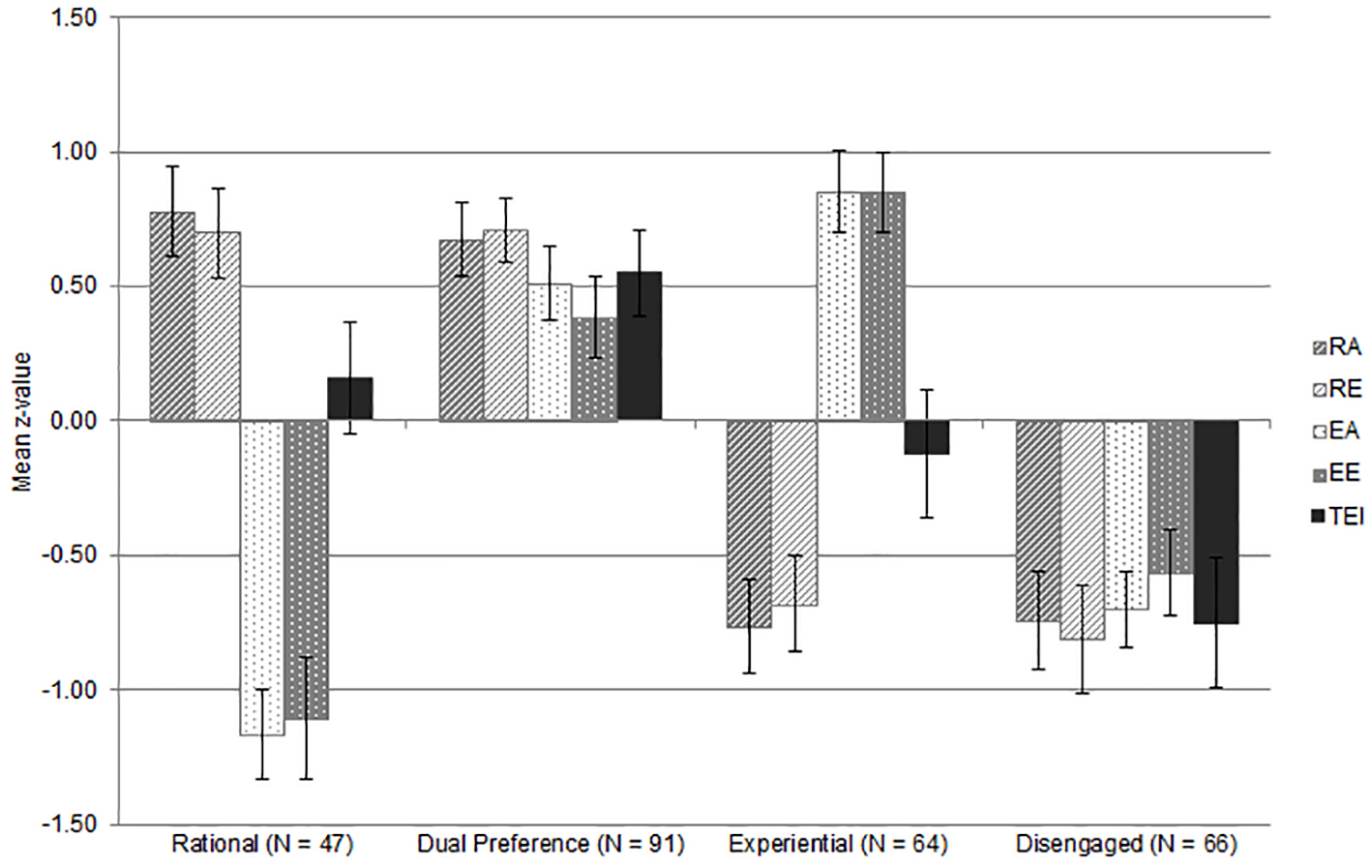
Standardized means and 95% confidence intervals for the four-profile solution. Numbers in parentheses indicate cluster size.

The LogLik, AIC and BIC values decreased with the increase in the number of latent groups, but seemed to plateau after the 4-profile solution (and even increase in case of BIC for the 6-profile solution), whereas entropy values were relatively high and similar for all solutions. Taking the number of participants per class into consideration, as well as theoretical expectations, we retained the solution with four latent profiles. We also examined the five- and six-profile solutions, but the additional profiles were merely extreme versions of already existing profiles from the four-profile solution. As shown in Figure 1, the four profiles corresponded to the rational, dual preference, experiential and disengaged profiles, previously identified by Fletcher et al. (2012). TEI was the most pronounced in the dual preference profile, and the least pronounced in the disengaged profile, while its standardized mean was essentially zero in both rational and experiential profiles. This indicates that the relationship of trait emotional intelligence with thinking styles is best understood when experientiality and rationality are observed in combination, rather than individually.

## Discussion

Regardless of perceiving themselves as more rational or more intuitive, most people recall at least one situation when their decision was based on affect even when knowing that the other option was “more rational”; or when they perceived an event as “just right”, though unable to explain it or provide rational reasons. However, it seems that some people rely on intuition more often than others, while some prefer logical thinking, regardless of context, or do not have a clear preference for any of these styles. From the perspective of classical personality models, it was meaningful to expect that some personality traits could predict the tendency towards intuitive vs. rational thinking, but past research showed that personality traits were not strong predictors of thinking styles – just like the Cognitive-experiential self-theory proposed (Epstein, 2003, 2016; Pacini & Epstein, 1999). CEST assumes that the way people process information relies on the experiential and rational learning systems – which are proposed as too specific and fundamental to be explained by other personality constructs, especially ES with its automatic, rapid and preconscious processes (Epstein, 2010). Still, we believe it is important to examine such intriguing and well-known constructs within the framework of other personality paradigms – in order to further extend the scientific knowledge about the human behavior through conceptual replications or applications of different models (e.g. exploring HEXACO as a predictor of rationality and experientiality, instead of Big Five traits), as well as through exploring new relations and possible predictors (e.g. trait emotional intelligence).

Although our study was not specifically designed to examine irrationality, we consider a deeper insight into constructs previously related to irrationality (Fletcher et al., 2012; Petrides et al., 2017) as having implications for the construct of irrationality itself. Can irrationality be understood through experientiality alone, without taking the rational dimension into account? Could TEI provide additional information about the preference for an irrational thinking style?

### Relations Between Constructs

Since the variables which correlated with rationality and experientiality also proved to be significant predictors of these thinking styles, we will discuss these analyses jointly, placing emphasis on the results of the regression analysis.

Similar to studies using the Big Five model (Pacini & Epstein, 1999; Witteman et al., 2009) we found that HEXACO personality traits predicted a small portion of the variance of rationality (31% vs. 37%, Pacini & Epstein, 1999, vs. 22%, Witteman et al., 2009). In our sample, most likely to rely on rational thinking were participants high in Openness to experience, Conscientiousness and to a certain degree Honesty/Humility. Emotionality and Agreeableness were not predictors of rationality, while eXtraversion was, but only when TEI was not included in the models.

These results are generally consistent with those of past studies (Pacini & Epstein, 1999; Witteman et al., 2009) and also conceptually expected, as self-perceived ability and engagement in complex problem-solving activities, characteristic of the rational thinking style, correspond well with those aspects of Openness to experience which refer to inquisitiveness and intellectual activities, as well as with organization and diligence aspects of Conscientiousness (Ashton & Lee, 2007). To our knowledge, our study is the first to establish a positive contribution of Honesty/Humility in explaining rationality, which may be interpreted in line with the view of the rational thinking style generally being associated with positively evaluated psychological traits (Pacini & Epstein, 1999). It should be noted, however, that this contribution is low, and the zero-order correlation was only obtained for the Rational Engagement subscale, suggesting that this result should be replicated.

Previous studies reported Neuroticism to have a negative contribution in predicting rationality, which was not obtained in our study. As already mentioned, HEXACO Emotionality and Big Five Neuroticism share only some of their facets, so this lack of a relationship with Emotionality is not entirely surprising. It is possible that changes in the structure of both Emotionality and Agreeableness have resulted in null correlations between rationality and these two dimensions in the HEXACO model, while correlations had been found for Big Five Neuroticism and (sometimes) Agreeableness.

On the other hand, the variance of experientiality remained essentially unexplained by HEXACO personality traits, which is not entirely in line with previous studies that reported low positive correlations with practically all Big Five traits (Pacini & Epstein, 1999; Witteman et al., 2009). As the current study is the first relating HEXACO with CEST constructs, it is unclear whether this pattern of results is a consequence of model differences or some sample characteristics. However, our results do confirm the thesis that intuitive, experiential and preconscious thinking processes are poorly explained by traditional personality topologies (Epstein, 2003, 2010). A recently proposed model of Disintegration as an additional basic personality trait reflecting psychosis proneness and including facets such as Enhanced Awareness and Magical Thinking (Knežević, Savić, Kutlešić, & Opačić, 2017) might fare better with regards to explaining experientiality. This idea, however, should be empirically tested.

We extended the findings regarding the variance of REI explained by basic personality traits by showing that TEI significantly predicts both rationality and experientiality, even after taking HEXACO personality traits into account. Moreover, TEI was the best predictor of experientiality and second-best predictor of rationality (after Openness for experience), indicating its importance for understanding preference for both of these thinking styles. These results are similar to those of Schutte et al. (2010), who measured emotional intelligence as an ability. Still, the contribution of this variable was moderate (5% for both thinking styles), meaning that individual differences in rationality and even more so in experientiality (which correlated to TEI to a lesser extent than rationality) cannot be reduced to classic personality traits, including TEI. The finding that experientiality remained largely unrelated to TEI is in line with the CEST assumption of preconscious automatic processes as an essential part of the evolutionarily old experiential system – related to but not reducible on affect-laden characteristics.

An interesting finding from the perspective of the TEI paradigm was that HEXACO eXtraversion lost its predictive power for rationality after TEI was included in the regression model. Even though eXtraversion and TEI are highly correlated, TEI proved more informative for the rationality construct, making eXtraversion a redundant predictor. This is fully in line with how the authors of the TEI model claim it should behave (Siegling, Vesely, et al., 2015). We have also found TEI to be positively associated with Agreeableness and Conscientiousness, which, along with the high correlation with eXtraversion, replicates previous findings, except that we did not find the negative correlation between TEI and Emotionality, which should be further tested (Veselka et al., 2010).

### Combined Thinking Style Dimensions and Their Relations to TEI

Looking at the data from a strictly correlational perspective, we observed that TEI was weakly positively related to experientiality and moderately to rationality, whereas the two thinking styles were independent of each other. Their distinction is further demonstrated by a differential pattern of relations with basic personality traits. This gives support to the dual view on thinking styles where rationality and intuition are seen as two orthogonal dimensions rather than two sides of the same coin (Wang et al., 2017).

However, CEST proposes that these dimensions can influence one another, and it is also expected that different combinations of high/low experientiality and rationality can produce differential adaptive outcomes (Epstein, 2003). Therefore, in order to better understand the relationship of TEI with CEST thinking styles, we performed a latent profile analysis aiming to uncover latent groups of participants with specific combinations of rational and experiential dimensions together with TEI. Our results replicated those of Fletcher et al. (2012) in that we also obtained a four profile solution with thinking style profiles being best described as rational, experiential, dual preference and disengaged. Moreover, TEI was sensitive to these profiles – dual preference (high experientiality and rationality) was followed by highest TEI scores. Disengaged participants, on the other hand, had the lowest TEI scores, while participants who primarily relied on rationality or experientiality did not differ in their TEI scores. In other words, both rational and experiential dimensions seem to be important for emotional intelligence and relying on one of them alone is not the characteristic of highly emotionally intelligent people – at least not when EI is conceptualized as the trait emotional self-efficacy.

TEI is conceptualized as a construct that collects variance related to affect-laden psychological processes, so its positive correlation with rationality (which was, in our sample, even stronger than with experientiality) might seem surprising. However, this is only so if rationality is considered isolated from the other CEST thinking style – experientiality. As we have already elaborated, the rational processes play an important role in emotional functioning by mediating the automatic influence of the experiential system on the rational one (Epstein, 2003, 2016). Therefore, the very processes of understanding and managing emotional functioning, proposed as an essential part of rationality within CEST, are inherent to the construct TEI.

In light of the discussion about irrationality, our results provide slightly more complex implications. Based on past findings that TEI directly negatively predicted irrationality (Petrides et al., 2017), our results imply that preferences for both experiential and rational thinking styles provide a good basis against irrationality, which is not in line with past findings that showed the lowest superstitions to be related to high rationality / low experientiality, and the highest superstitions to high experientiality / low rationality thinking styles (Fletcher et al., 2012). However, the percentage of variance shared between REI and TEI was only moderate in our study and, moreover, the shared variance was not necessarily related to irrationality. On the other hand, Fletcher et al. (2012) used a specific superstition scale, so the implications of their results for the general construct of irrationality should also be further tested.

From the CEST perspective, it is worth remembering that although ES is a source of irrationality, it is also proposed as an evolutionarily old system, essential for survival (Epstein, 2016). A recent meta-analysis demonstrated that although intuition is often considered “irrational” and less accurate than rational thinking, in certain complex or subjectively evaluated situations decisions based on intuition were more successful (Phillips et al., 2016). Accordingly, CEST assumes that the adaptive response is not to suppress the influence of ES, but to understand it and learn the way to correct it when it is wrong or inadequate, where RS has an important role (Epstein, 2003, 2016). In other words, even though high experientiality reflects the tendencies towards superstitions and irrational thinking, if the rational dimension is also high, there is a possibility for a person to overcome irrational beliefs. In contrast, if rationality is low, irrational beliefs are less likely to be replaced by more rational and logical thinking. Since our study design did not include specific criterion variables to measure irrationality/superstitions, it was not possible to test these assumptions directly. Future research should reveal more detail about the relation between irrationality and specific combinations of rational and experiential styles, as well as about the way people approach the world when they perceive themselves as poor on both thinking styles.

### Study Limitations

As instrument validation was not the primary goal of our study, we did not assess the structural validity of either REI or TEIQue-SF. Future studies should validate the Serbian versions of these instruments, preferably on a larger sample.

It is important to note that the experiential dimension has been further theoretically and empirically developed with three factors: intuition, emotionality, and imagination (Norris & Epstein, 2011). In our study REI-40 was employed as an original and widely used instrument, also validated in other languages (e.g. Slovakian: Ballová Mikušková, Hanák, & Čavojová, 2015; Swedish: Björklund & Bäckström, 2008), although not previously validated in Serbian. We assumed that REI-40 is appropriate for our study design, but in order to further examine the possible predictors of the experiential dimension it is still recommended to do this separately for the three experiential dimension factors as criteria variables.

We chose to use only the global TEI score, since the use of subscales did not bring any additional information to the analyses. However, it is possible that factors (and facets) of the full-length instrument could provide more specific information on the TEI-REI relationship, which should be further investigated.

Finally, even though we did not observe a restriction of range for any of the scales, participants in our study were drawn from a student population. Furthermore, our sample was gender-imbalanced and the number of male participants was too small to warrant separate analyses by gender. Therefore, future studies should replicate these results in more representative samples.

### Conclusion

Our study contributes to the accumulation of knowledge about personality space, showing that personality constructs such as trait emotional intelligence and rational and experiential thinking styles could explain the unique variance of complex psychological processes, beyond those provided by classical personality models. Moreover, relating constructs from different personality paradigms opens possibilities for a better understanding of complex phenomena, such as the way we process information about the world and ourselves. Finally, our results support the position that searching for possible predictors and adaptive consequences of not only rational and experiential thinking styles separately, but also of their specific combinations is worthwhile.
